# Multidimensional Research on Hair Loss in Young Chinese Females With Oily Scalps

**DOI:** 10.1111/jocd.70426

**Published:** 2025-09-07

**Authors:** Siqi Shao, Benyue Li, Jie Yang, Fengwei Qi, Qi Liu, Fengnian Zhao

**Affiliations:** ^1^ School of Light Industry Science and Engineering Beijing Technology and Business University Beijing People's Republic of China; ^2^ Shandong Huawutang Biotechnology Co., Ltd Jinan People's Republic of China; ^3^ Beijing EWISH Testing Technology Co., Ltd Beijing People's Republic of China

**Keywords:** hair loss, lipid metabolism, oily scalps, scalp microbiome, young females

## Abstract

**Background:**

In recent years, the problem of female alopecia has been increasing and has shown a trend toward youthfulness. However, there are fewer studies on young female alopecia in the existing literature.

**Aim:**

We aimed to study the possible causes of hair loss in young Chinese females aged 18–35 with oily scalps.

**Method:**

Scalp physiological parameters were measured by non‐invasive measurement techniques, scalp lipids were measured by UPLC‐MS/MS, scalp microbiota were measured by macro‐genome sequencing, and the results were analyzed in a multidimensional combined analysis.

**Result:**

The problematic population showed significant differences in several scalp physiological indices, lipid composition, and microbial community structure compared with the normal population, and eight closely related differential lipids, as well as microorganisms with high relative abundance at the phylum and genus levels, were screened.

**Conclusion:**

Abnormalities in specific lipids and metabolic pathways, as well as microbiological changes, are associated with hair loss and oily scalp problems.

## 1 Introduction

In recent years, hair loss has become an increasingly common and serious problem for human beings, which is more prominent in men with androgenetic alopecia (AGA) [1]. AGA is a polygenic hereditary non‐scarring alopecia with typical features of gradual miniaturization of the hair follicles at the ends of the head, which is mainly influenced by androgens among many other influencing factors, with dihydrotestosterone (DHT) produced by the enzyme testosterone 5α reductase thought to be an important contributing factor [1, 2, 3, 4]. However, the problem of hair loss in females also should not be ignored, which has produced a range of negative effects on patients' quality of life, self‐esteem, stress, depression, and other psychosocial aspects [5, 6, 7, 8].

The most common type of hair loss problem among females is female pattern hair loss (FPHL), which is now mainly used to denote androgenetic alopecia in females since the relationship between female hair loss and androgens is unclear [9, 10]. FPHL is a diffuse non‐scarring alopecia with clinical features of a preserved frontal hairline but thinning hair [11, 12]. The pathogenesis of FPHL is not well defined and is currently thought to be related to various factors such as genetic susceptibility, androgens, non‐androgenic factors, environmental factors, and microinflammation [10, 13, 14]. Previous studies have shown that the prevalence of FPHL increases with age, especially in females over 50 years of age [15, 16]. However, it has been found that the average age of females suffering from hair loss gradually decreases, showing a rejuvenation trend in recent years [17, 18]. Up to now, there has been limited research on hair loss in females, especially young females, making the pathogenesis for hair loss in young females still unclear. Therefore, it is of great significance to investigate the problem of hair loss in young females and explore the potential mechanisms and factors.

Some clinical studies have shown that seborrheic dermatitis, with greasy scalp as a common symptom, is the most common associated condition in patients with FPHL [17, 19], which suggests that abnormalities in scalp lipid metabolism and its further triggering of disorders in the scalp microenvironment may be closely related to the problem of hair loss. It has also been shown that in young females, the severity of female‐pattern hair loss is directly proportional to the level of scalp oil secretion [19], FPHL evolves from the gradual shortening of the hair anagen phase and miniaturization of the hair follicle [9], whereas the excessive secretion of scalp oil may lead to a change in the environment of the follicle and further disrupt the skin barrier within the follicle and cause inflammation [20]. Scalp microbes have also been shown to play an important role in maintaining skin homeostasis and suppressing scalp inflammation [21], and when the balance of the microbial community is disrupted, changes in composition can lead to a variety of scalp disorders [22].

Based on the above research, we chose young Chinese females between 18 and 35 who have the problem of both hair loss and greasy hair as the study subjects, and used young females in the cities of Beijing and Hangzhou as representative samples for the study. In this study, a multidimensional joint analysis method was used to compare the scalp physiological indexes, scalp lipids, and scalp microorganisms of young females in the experimental and control groups. The study focused on the scalp differences between the problematic and healthy populations with both hair loss and oily scalp problems, and further investigated the possible causes of alopecia and greasy hair in females of the 18–35 age group by analyzing the differences in scalp lipids and scalp microorganisms between the different groups and their potential impact on the scalp physiological indexes. This study aims to provide a reference for the study of scalp problems in young females and hopefully will offer new perspectives and a scientific basis for future research.

## 2 Methods

### 2.1 Subjects

This study was conducted with 120 Chinese female subjects aged 18–35 with a combination of both hair loss and greasy hair, as well as 120 Chinese females with healthy scalps. We selected participants who exhibited high levels of scalp oil production, were troubled by excessive hair loss, and had a hair loss count greater than 10 out of 60 combing methods. The exclusion criteria include pregnancy, having received treatment for scalp diseases, and having had hair care or perming and dyeing within the last month or BMI > 27.

### 2.2 Study Design

A cross‐sectional controlled study based on human test data and questionnaires was adopted. For experimental purposes, subjects were asked not to wash their hair for 48 ± 4 h before each scalp collection and not to comb their hair on the day of collection.

Before measurement, subjects were required to wait 30 min in an environment with constant temperature and humidity. During this time, they were required to complete a self‐assessment questionnaire on scalp and hair loss. Next, we divided subjects into four groups based on scalp condition and geographic location: the experimental and control groups in Hangzhou and Beijing, respectively, and then performed the collection of scalp physiological indicators, scalp lipids, and scalp microbiota of the subjects.

### 2.3 Measurement of Scalp Physiological Factors

In this study, the scalp physiological factors include the moisture content of the stratum corneum, transepidermal water loss (TEWL), skin pH value, skin oil content, overall hair density, and the number of hair loss. According to the method of dividing the scalp into 12 zones from the forehead to the nape of the neck, 6 of these more visible areas were selected as test areas. The detailed information is shown in Figure S1 and Table S1.

The moisture content of the stratum corneum was measured in the test area within 2 cm × 2 cm using the probe moisture tester Hydration (CORTEX TECHNOLOGY, Denmark). TEWL was also measured in the test area within 2 cm × 2 cm using a Tewameter TM Nano (Courage & Khazaka, Germany). Skin pH value was measured using a Skin‐pH‐Meter PH 905 (Courage & Khazaka, Germany). Skin oil content was measured using a Meibometer MB 560 (Courage & Khazaka, Germany). The detailed procedures are described in the Data S1.

The overall hair density assessment consisted of two parts: a visual assessment and an image assessment. The density of hair on the top of the subject's head was first visually assessed and recorded by a licensed dermatologist on‐site under the constant light from fluorescent tubes or LED light with a color temperature of 5500–6500 K, on a 0–7 scale (Table S2). Next, images of the top of the subject's head were captured at a fixed height and position parallel to the top of the head using a digital single‐lens reflex (SLR) camera 850D from Canon in Japan. Then, at the end of the field collection, a dermatologist assessed the hair density of the photographs taken according to the grading criteria and recorded the scoring grade.

Before counting the number of hair loss, subjects should wait in a constant temperature and humidity environment for 30 min to acclimatize to the environment. Measurements were taken by the same trained technician using the same size comb. The technician combed the subjects' hair 60 times (30 times left and 30 times right) at a constant speed, following the sequence from left front to left back and right front to right back. The hair shed by the subjects during these 60 combing sessions was collected at the end of the measurements, and the shed hairs that were whole and contained hair follicles were counted.

### 2.4 Measurement of Scalp Lipid

The scalp lipid samples were obtained as follows. First, select a hair seam in the collection area, and using hands and a comb, try to open up the seam and depress the hair on both sides. Then, a piece of sticker tape was placed over the open hairline with tweezers, and the tape was pressed with a pressure bar for 20 s, ensuring that all of the tape was within the pressure range of the bar during this time. When the time comes, use tweezers to remove the tape and place it in the EP tube, paying attention to placing it in such a way that the tape in the tube does not overlap or become entangled as much as possible. Next, the same procedure was followed to complete lipid collection from two hair seams in each region, for a total of 24 seams. Immediately after completing the collection, each tube was placed in dry ice for cryopreservation.

The collected lipid samples were processed using an extraction solvent containing an internal standard mixture (MTBE: MeOH = 3:1, v/v), ultrapure water, and lipid complex solution. The sample extracts were then analyzed using an LC‐ESI‐MS/MS system, and raw lipid data were derived. Specific steps for lipid sample processing and analysis are detailed in the Data S1.

### 2.5 Measurement of Scalp Microflora

The scalp microflora samples were collected as follows. First, a comb was used to separate the subject's hair along the midline, dividing it roughly equally into 12 regions (6 on the left and 6 on the right), and clips were used to hold the hair in each region. Next, within the above‐mentioned area, select the hair seams that have not undergone lipid collection for collection. Using a swab completely moistened with saline at a 45° angle of inclination, smoothly and slowly swab a 5‐cm hairline in one of the selected areas 20 times. Fracture the swab into the tube after wiping is complete. Then, choose 2 hair seams in each region to carry out the above operation. The operation needs to ensure that each area of the collection technique and the number of friction is consistent. Immediately after completing the collection of each tube, it was frozen in liquid nitrogen for storage.

After the collection of scalp microflora, the raw samples that have been tested and qualified will be used for library construction, and the constructed library will be sequenced using Illumina PE150 to obtain the raw data (Raw Data). After obtaining the Raw Data, we processed it with fastp software, MEGAHIT assembly software, Bowtie2 software, MetaGeneMark predicted open reading frames (ORFs), and CD‐HIT software, and finally obtained the Unigenes, which were further processed by DIAMOND software and LCA algorithms, and then obtained the corresponding species annotation information. Detailed parameters and specific procedures for the above processing and analysis of microbial results are described in the Data S1.

### 2.6 Data Analysis

Statistical processing was performed using SPSS 27.0 system software. The t‐test was used to determine the differences between the different scalp parameters in the experimental and control groups. Statistical significance was determined as *p* < 0.05. During lipid data processing, lipid initial data were log‐transformed (log2) and mean‐centered for OPLS‐DA analysis using the R software package MetaboAnalystR. Multiple linear regression analysis and partial least squares analysis of scalp physiological indices and screened differential metabolites were performed using SPSS 27.0 and smartPLS, respectively. During microbiological data processing, Qubit 4.0 was used to quantify the constructed libraries, then Qsep400 was used to test the libraries, and Illumina PE150 sequencing was performed on the different libraries after passing the quality control.

## 3 Results

### 3.1 Analysis of Scalp Physiological Indicators

**FIGURE 1:**
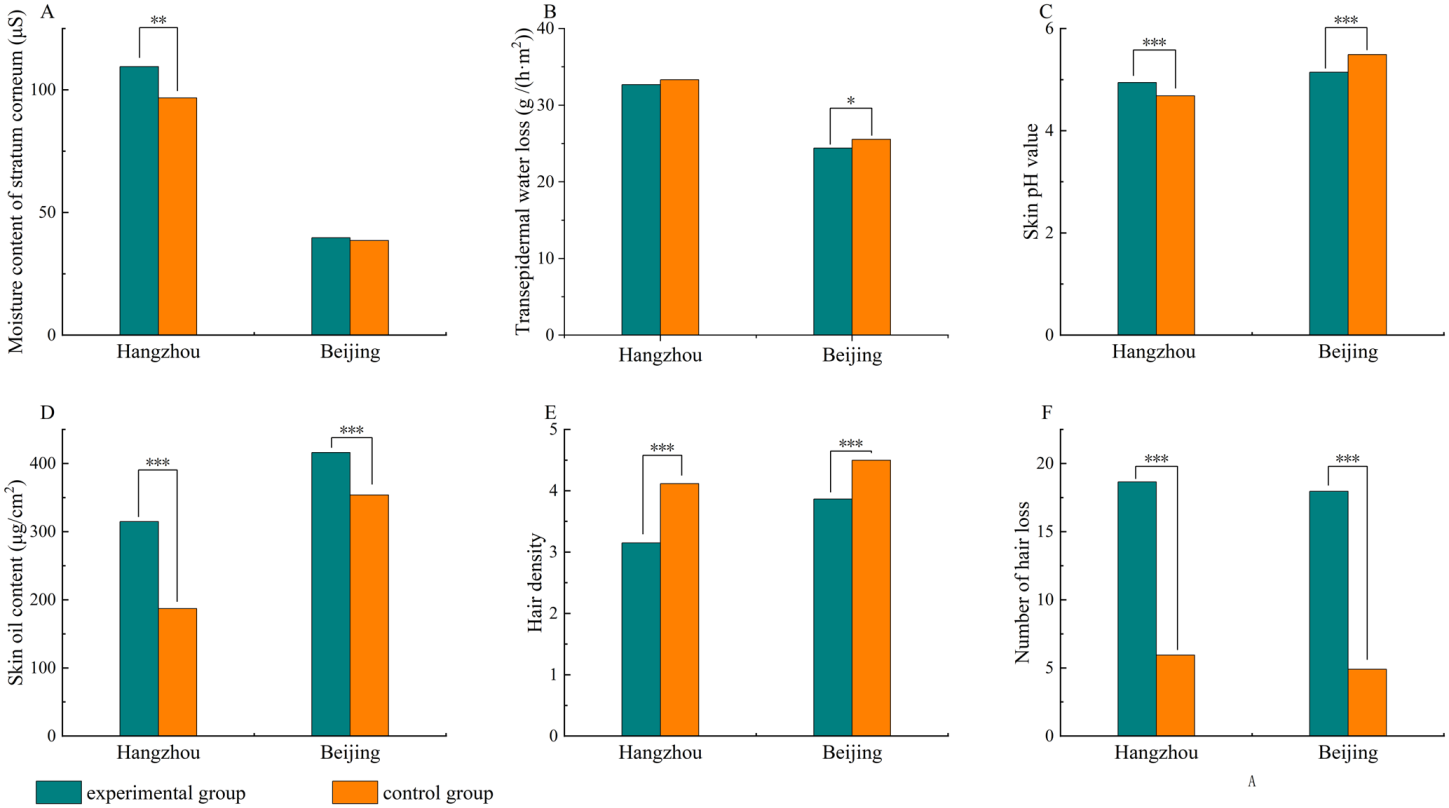
Scalp physiological indicator data. (A) Mean values of moisture content of stratum corneum in Hangzhou and Beijing subjects. (B) Mean values of TEWL in Hangzhou and Beijing subjects. (C) Mean values of skin pH in Hangzhou and Beijing subjects. (D) Mean values of skin oil content in Hangzhou and Beijing subjects. (E) Mean values of hair density in Hangzhou and Beijing subjects. (F) Mean values of hair loss count in Hangzhou and Beijing subjects. ***p < 0.001; **p < 0.01; *p < 0.05.

By analyzing data from different scalp physiological indicators, we found that the moisture content of the stratum corneum in Hangzhou and the TEWL in Beijing showed significant differences between the problematic and normal populations (Figure 1A,B). Meanwhile, there were significant differences in skin pH, skin oil content, hair density, and hair loss counts between problematic and normal populations in both Hangzhou and Beijing (Figure 1C–F). The trends of skin oil content, which can visualize the degree of oily scalps, as well as hair density and number of hair loss, which can reflect the degree of hair loss, were consistent across both regions. In contrast, the trends of scalp pH value were different between the two regions, which might be affected by local environmental factors and could not intuitively reflect the degree of hair loss and oily scalps.

### 3.2 Differences in Scalp Lipid Composition

To further investigate the differences in scalp lipids between the target and control populations, we detected 676 lipids in the subject samples by the UPLC‐MS/MS assay platform extensively targeted metabolomic technology. Herein, the Orthogonal Partial Least Squares Discriminant Analysis (OPLS‐DA) was used to differentiate groups based on metabolic profiles. OPLS‐DA analysis is a multivariate statistical analysis method with supervised pattern recognition, which effectively excludes study‐irrelevant effects and thereby screens for differential metabolites. After normalizing the scalp lipid data from Beijing and Hangzhou, we constructed an OPLS‐DA model and used OPLS‐DA to plot scores for the Hangzhou experimental group (i.e., WT‐HZ), Hangzhou control group (i.e., ZC‐HZ), Beijing experimental group (i.e., WT‐BJ), and Beijing control group (i.e., ZC‐BJ) in pairwise analysis. In this model, *Q* ^2^ indicates the predictive ability of the model, and *Q* ^2^ of all comparison groups (WT‐BJ vs. ZC‐BJ and WT‐HZ vs. ZC‐HZ) is greater than 0.5 (Figure S2A,B), indicating that the constructed model is valid. OPLS‐DA score plots show significant separation occurs in different comparison groups (Figure S2C,D), indicating obvious differences between groups.

**FIGURE 2:**
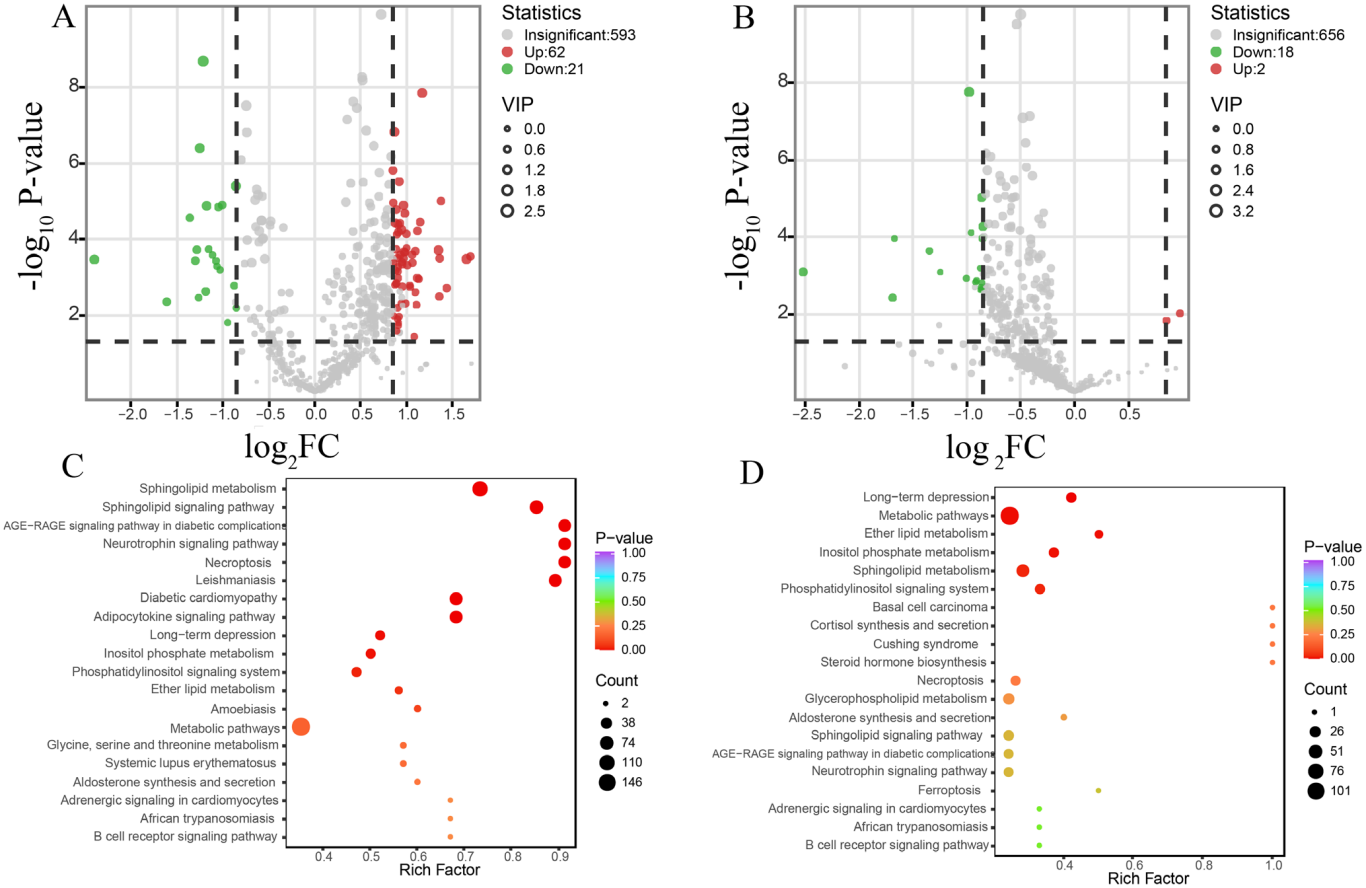
Volcano maps of differential metabolites and maps of enriched metabolic pathways. (A) Volcanograms of WT‐BJ (Beijing experimental group) and ZC‐BJ (Beijing control group). (B) Volcano diagrams of WT‐HZ (Hangzhou experimental group) and ZC‐HZ (Hangzhou control group). (C) Differential lipid pathway enrichment maps of WT‐BJ and ZC‐BJ. (D) Differential lipid pathway enrichment map of WT‐HZ and ZC‐HZ.

After establishing the grouping, to initially find out which were the differential metabolites among the detected lipids, we screened them using Fold Change (FC), Variable Importance in Projection (VIP), and *p*‐value, and regarded the lipid metabolites that simultaneously satisfied both FC > 1.8, VIP > 1, and *p*‐value < 0.05 as differential metabolites. The results showed that there were 83 differential metabolites, of which 62 were up‐regulated and 21 were down‐regulated in the comparison between the WT‐BJ and ZC‐BJ groups, and 20 differential metabolites, of which 2 were up‐regulated and 18 were down‐regulated in the comparison between the WT‐HZ and ZC‐HZ groups (Figure 2A,B).

To further identify more relevant differential lipids, a multiple linear regression analysis was first performed using SPSS with the 103 lipid components selected as independent variables and scalp physiological indices as dependent variables to observe the relationship and based on the results to exclude the lipids that did not have a significant relationship with any of the six selected scalp physiological indices. By this step, we further selected 72 lipids.

We then performed Partial Least Squares (PLS) analysis using smartPLS software to construct a path model with 6 scalp physiological indices and 72 lipids as latent variables, and the PLS algorithm was used to obtain the analytical results. Based on the results of our analysis, we focused on the top 20 lipids with the largest absolute values of the path coefficients in the data corresponding to scalp lipid content and hair loss counts, and ultimately filtered out eight lipids with strong relationships to both scalp physiological metrics, namely TG (16:0_18:2_18:3), Cer (d16:1/18:0), Cer (d18:1/17:0), DG (18:1_20:3), Cer (d16:1/24:0), Cer (d16:1/24:1), Cer (d16:1/26:1), and DG (18:0_22:0).

Matching all the differential metabolites in the different comparison groups to KEGG's database to obtain information on the pathways in which the metabolites are involved, and then performing enrichment analysis on the annotated results to obtain the pathways in which the differential metabolites are more enriched. Differential metabolites in the WT‐BJ and ZC‐BJ groups were mainly annotated and enriched in the sphingolipid metabolic pathway, the neurotrophin signaling pathway, and the adipocytokine signaling pathway; differential metabolites in the WT‐HZ and ZC‐HZ groups were mainly annotated and enriched in the sphingolipid metabolic pathway, the phosphatidylinositol metabolic pathway, and the ether lipid metabolic pathway. Some metabolic pathways overlap in these four comparison groups, such as the sphingolipid metabolic pathway (Figure 2C,D).

### 3.3 Microbial Colony Differences

**FIGURE 3:**
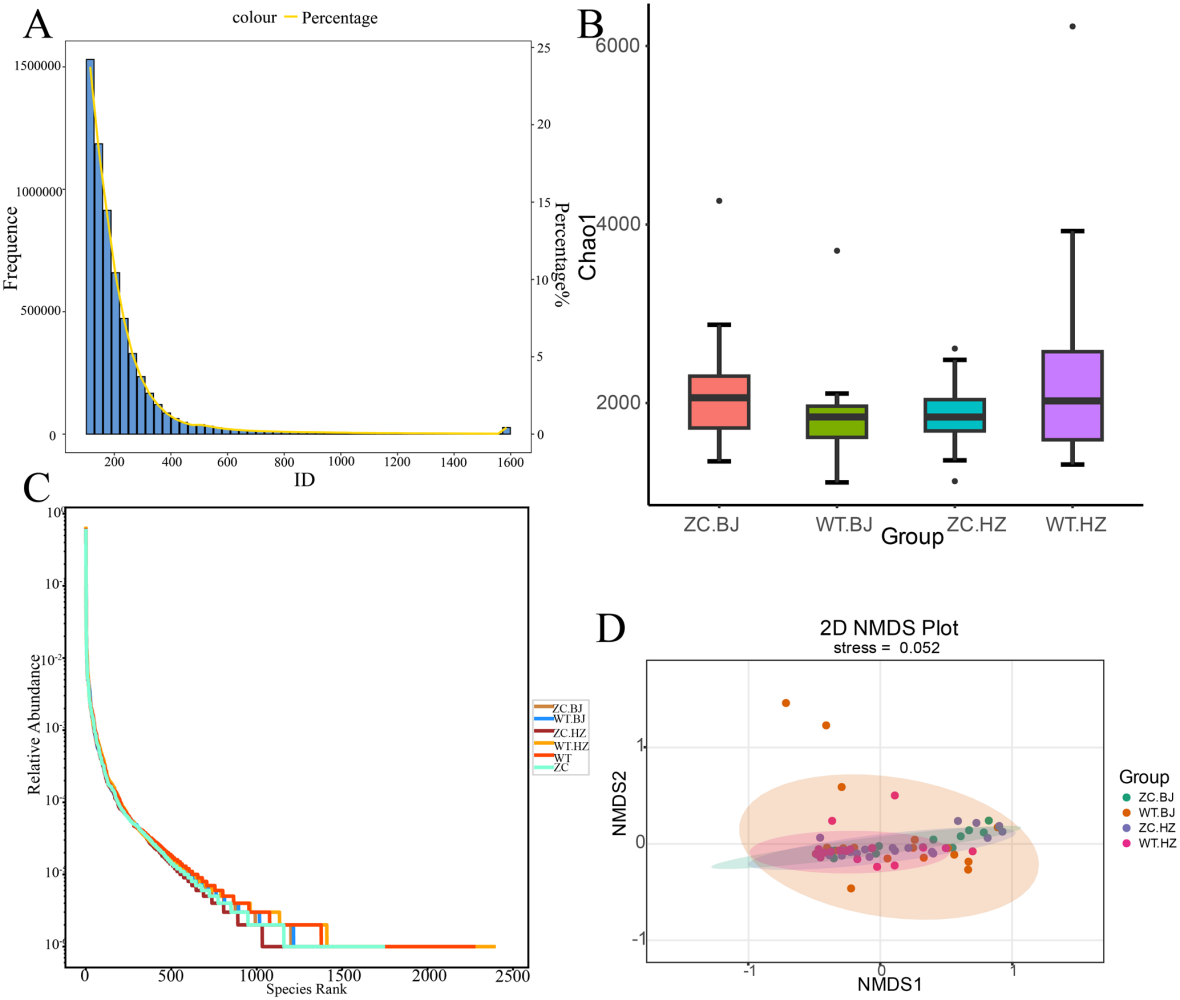
(A) Statistical plot of the Unigenes length distribution. (B) Hierarchical clustering curves based on species abundance. (C) Hierarchical clustering curves based on species abundance. (D) NMDS results for species based on the gate level.

To further investigate the differences in scalp microbiology between the experimental and control groups, we used macro‐genome sequencing technology for the study. Based on Illumina high‐throughput sequencing, we obtained 6.14 million valid sequences from 78 samples, and the average length of the sequences was about 227.9 bp (Figure 3A).

#### 3.3.1 Analysis of Scalp Microbial Community Diversity

Alpha Diversity reflects the richness and diversity of microbial communities within a sample and is commonly represented by the Chao1 and Shannon indices. The mean values of the Chao1 index and Shannon index for the WT‐HZ group were 2356.3937 and 2.0491, while those for the ZC‐HZ group were 1850.4687 and 1.9741. And the mean values of the Chao1 index and Shannon index for the WT‐BJ group were 1907.6163 and 2.009, while those for the ZC‐BJ group were 2124.0459 and 1.9266. The t‐test analysis showed that there was a significant difference in the Chao1 index between the WT‐HZ and ZC‐HZ groups (*p* < 0.05), while no significant difference was found in Beijing (*p* = 0.15 > 0.05), indicating that the number of microbial species varied greatly among samples from different regions (Figure 3B).

In addition, the larger the Channon index, the greater the uncertainty, and the higher the community diversity. The mean values of both WT‐HZ and WT‐BJ groups were larger than those of ZC‐HZ and ZC‐BJ groups, indicating that the diversity of the microbial community in WT‐HZ and WT‐BJ was larger than that in ZC‐HZ and ZC‐BJ. We plotted hierarchical clustering curves to visualize these differences more (Figure 3C). It can be observed that there is a significant difference between WT‐HZ and ZC‐HZ in the width of the curves, with the WT‐HZ group having wider curves, indicating higher species richness, while the differences between WT‐BJ and ZC‐BJ are not so obvious.

Beta diversity can be used to reflect species differentiation among microbial communities in a sample. NMDS dimensionality reduction analysis, a metric‐free multidimensional calibration method, is a nonlinear model based on the Bray‐Curtis distance for analysis to overcome the shortcomings of PCA and PCoA. From the results of the NMDS analysis based on the gate level (Figure 3D), it can be seen that there is a certain separation trend between the WT‐BJ group and the ZC‐BJ group, indicating that there are some differences in the microbial community structure between these two groups. The graph did not separate the WT‐HZ and ZC‐HZ groups, which indicated that these two groups had a high similarity in microbial community structure. In this analysis, stress equal to 0.052 (Figure 3D) is less than 0.2, which indicates that the results of this analysis have some reliability.

#### 3.3.2 Analysis of Microbial Species Composition and Abundance

**FIGURE 4:**
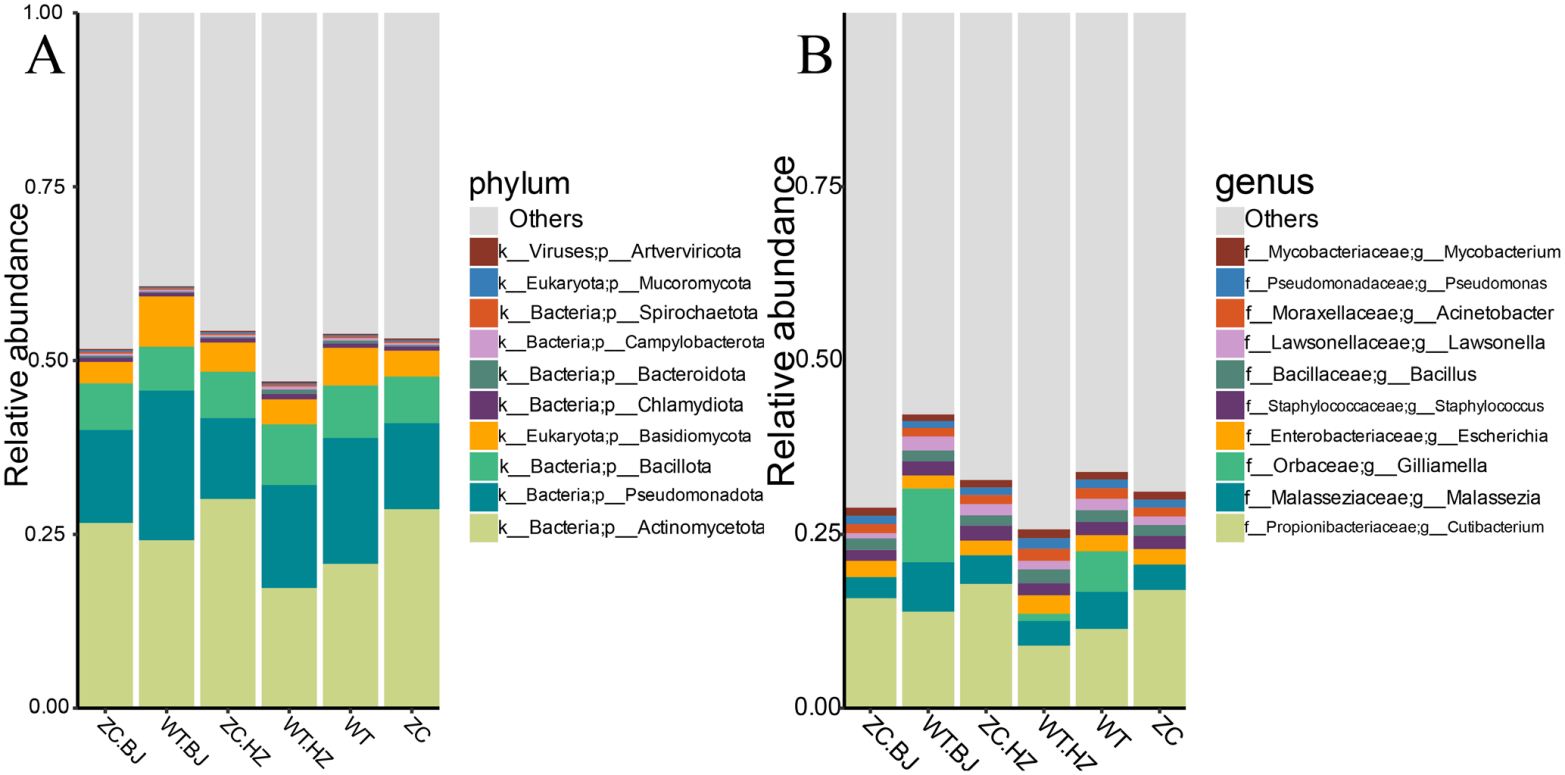
Analysis of different levels of differences in scalp microorganisms between different groups. (A) The relative abundance of different species of scalp microbial communities at the phylum level. (B) The relative abundance of different species of scalp microorganisms at the genus level (WT is the combined result of the experimental group in Beijing and Hangzhou, and ZC is the combined result of the control group in Beijing and Hangzhou).

According to the results of species annotation, at the phylum level (Figure 4A), the main dominant phyla in the scalp microbial community in Hangzhou and Beijing were *Actinobacteria*, *Pseudomonas*, *Bacillota*, and *Basidiomycota*. In the experimental group in Beijing, compared with the control group, the relative abundance of *Actinobacteria* and *Bacillota* decreased, and the relative abundance of *Pseudomonas* and *Basidiomycota* increased. Comparing the experimental group in Hangzhou with the control group, the relative abundance of *Actinobacteria* and *Basidiomycota* decreased, and the relative abundance of *Pseudomonas* and *Bacillota* increased.

At the genus level (Figure 4B), *Cutibacterium*, *Malassezia*, and *Escherichia* were the dominant genera in the scalp microbial communities of the different subgroups in Hangzhou and Beijing, and a comparison of the experimental and control groups in the two regions showed that *Gilliamella* was detected in theexperimental group but not in the control group. In addition, the relative abundance of *Cutibacterium* in the experimental and control groups in Hangzhou decreased significantly, the relative abundance of *Malassezia* decreased, and the relative abundance of *Escherichia* increased. In the experimental group in Beijing, the relative abundance of *Cutibacterium* decreased, the relative abundance of *Malassezia* increased, and the relative abundance of *Escherichia* decreased compared with the control group.

#### 3.3.3 Analysis of Microbial Community Differences Between Groups

To study the species with significant differences between groups, starting from the species abundance tables of different tiers, the *p*‐value was obtained by hypothesis testing of the species abundance data between groups through the Metastats method, then the q‐value was obtained by correcting the *p*‐value, and finally, the species with differentiation were screened according to the *q*‐value. According to the results of Metastatic analysis (Figure S3A–J), at the gate level, *Actinomycetota*, *Bacteroidota*, *Campylobacterota*, *Thermotogota*, *Chytridiomycota*, *Zoopagomycota*, *Hofneiviricota*, *Kitrinoviricota*, and *Pisuviricota* could be screened between the experimental and control groups in Hangzhou, which are microbial species with significant differences. Beijing's experimental and control groups can be screened for *Fusobacteriota* and *Hofneiviricota*.

**FIGURE 5:**
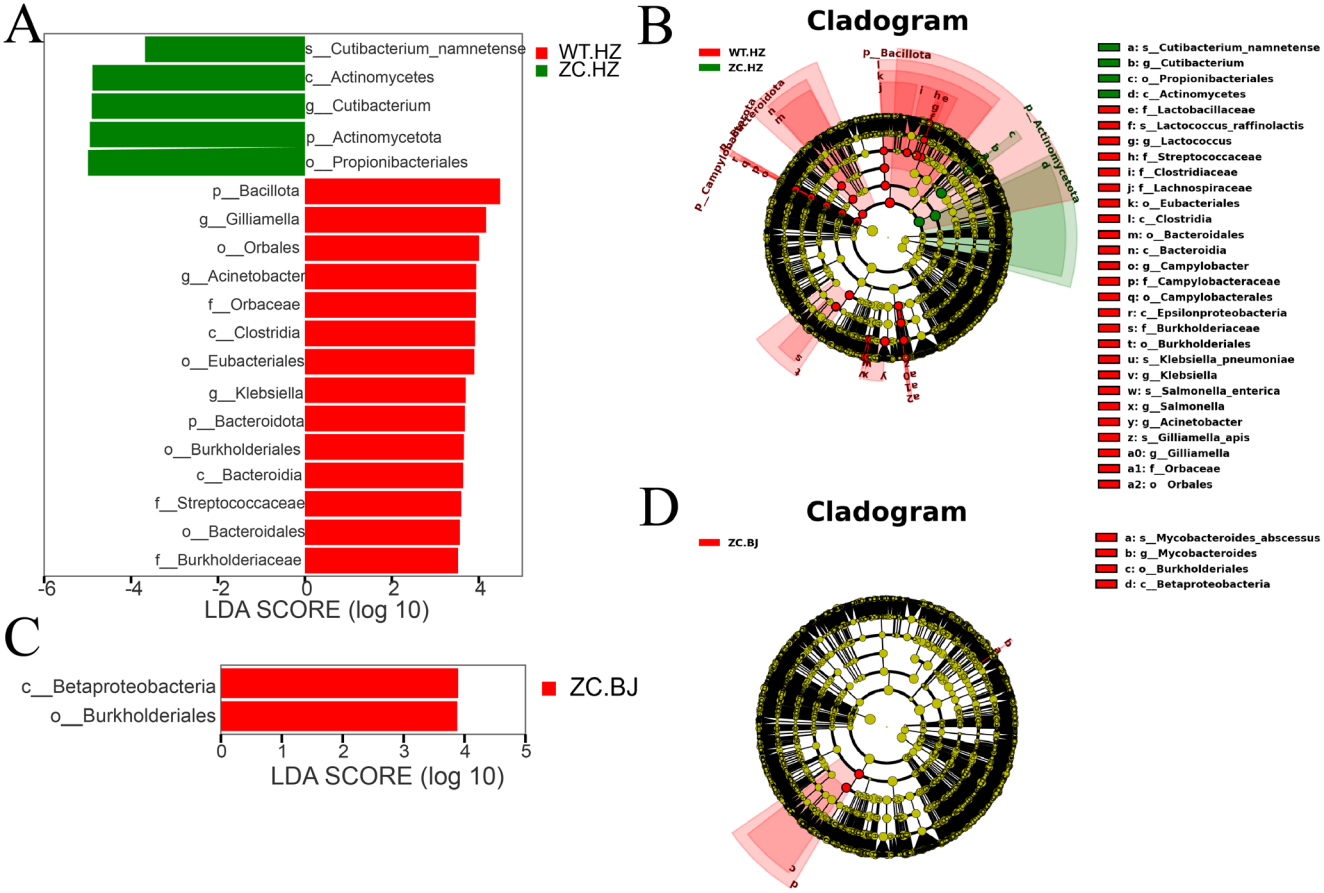
LefSe analysis for differential microorganisms. The distribution of (A) LDA values and (B) evolutionary branching diagram of differential microorganisms in Hangzhou. The distribution of (C) LDA values and (D) evolutionary branching diagram of differential microorganisms in Beijing.

Biomarkers of species with significant differences in different groups were further screened by LEfSe analysis. In the histogram of LDA value distribution, we considered the species with an LDA Score value greater than 3.5 to be statistically different biomarkers between groups. The red and green nodes in the figure represent microorganisms that play significant roles in the experimental and control groups, respectively. After analyzing the experimental and control groups in Hangzhou (Figure 5A,B), it was found that *Gilliamella*, *Acinetobacter*, and *Klebsiella* were detected at the genus level with high relative abundance in the WT‐HZ group, and *Cutibacterium* was detected with high relative abundance in the ZC‐HZ group, which may have an important influence on the microbial communities of these two groups, respectively. After analyzing the results from Beijing (Figure 5C,D), it was found that *Burkholderiales* was detected at the order level in the ZC‐BJ group with a higher relative abundance, which may have an important influence on the microbial community of this group, and the corresponding microorganisms were not detected in the WT‐BJ group.

### 3.4 Joint Analysis of Scalp Microbiome and Scalp Lipid Metabolism

To investigate the correlation between scalp microbiota and scalp lipid metabolism, the present study was based on the results of macro‐genomics and metabolomics, and the screening of differential lipids and scalp microbes was jointly analyzed. Based on the Pearson correlation, the correlation clustering analysis of differential microorganisms and differential metabolites at the genus level (Figure S4A,B) indicated that the differential microorganisms and metabolites were highly correlated in both Beijing and Hangzhou. Based on the criteria of correlation coefficient |*r*| ≥ 0.4 and *p*‐value of correlation coefficient significance test < 0.05, the top 50 pairs of significantly correlated differential microorganisms and differential metabolites were screened out and plotted as chordal plots (Figure S4C,D). It can be concluded that, at the genus level, the top five groups of scalp metabolites with the strongest correlation with scalp microorganisms screened in Beijing were TG (16:0_18:0_18:0) and *Methylobacter*, *Pustulibacterium*, *Tatlockia*, *Paludibacter*, and *Candidatus_Cyrtobacter*. In Hangzhou, the top five groups for scalp metabolites and scalp microbial screening were Cer (d18:2/18:0(2OH)) and *Kitasatospora*, *Porphyromonas*, Cer (d20:1/23:1) and *Oceanicella*, *Rhizorhapis*, and *Tequintavirus*. It is also noteworthy that in the joint analysis, we found that Cer (d19:1/18:0) was negatively correlated with *Kitasatospora* and *Dorea*, and DG (18:0_22:0) was negatively correlated with *Bacterioplanes*, *Lacrimispora*, and *Dorea*.

## 4 Discussion

By analyzing the experimental results, we found some differences in scalp lipid composition and scalp microbiota between problematic and normal populations, which may lead to changes in scalp physiological parameters and further impact hair loss and oily scalp problems. In this study, we screened eight lipids that are closely related to both hair loss and oily scalp problems and also identified two lipid metabolic pathways that are closely related to both hair loss and oily scalp problems in young Chinese females by KEGG‐enriched pathway analysis. Meanwhile, the composition of the scalp microbial community was analyzed at the phylum level and genus level, and the dominant phylum and genus were screened out, and statistically significant biomarkers were screened out by Metastats and LEfSe analyses, which may have a significant impact on the scalp microbial community.

### 4.1 Scalp Differential Lipids and Metabolic Pathways

The eight lipids screened can be categorized into three groups: ceramides, triglycerides, and diglycerides. Ceramides are central to the structure and function of the epidermal permeability barrier and play an important role in the induction of apoptosis [23, 24]. Elevated ceramide levels in cells are one of the hallmarks of early apoptosis [25], and it is inferred that abnormal accumulation of ceramides in the hair follicle may adversely affect scalp cells, which in turn may cause scalp health problems. Different types of ceramides have different properties, for example, Cer(d16:1/18:0) is associated with cell growth, Cer(d16:1/24:0) affects the characterization and reorganization of cell membranes, and Cer (d16:1/24:1) has been linked to cell signaling and cell growth [26, 27, 28, 29]. These properties may have different effects on hair loss and oily scalp problems. Triglycerides and diglycerides are both components of human sebum [30, 31] and are directly related to scalp oil secretion. For example, DG(18:0_22:0) and DG(18:1_20:3) play an important role in cellular signaling and intermediates of lipid metabolism [32], which may have an impact on signaling in scalp cells and lipid metabolism in the scalp. In addition, the relationship between Cer(d18:1/17:0), Cer(d16:1/26:1), and TG(16:0_18:2_18:3) and hair loss and oily scalp problems remains to be elucidated and can be investigated in the future studies.

In addition to screening eight lipids closely related to hair loss and oily scalp problems, we also found two closely related lipid metabolic pathways by KEGG enrichment pathway analysis. Among them, the sphingolipid metabolic pathway not only has a structural function as one of the major components of cell membranes, but also involves the synthesis and catabolism of a variety of lipid‐active molecules, such as ceramides, sphingomyelins, and ceramide‐1‐phosphate (S1P), and is closely related to processes such as cell signaling and cell growth regulation [33, 34]. In addition, the sphingolipid metabolic pathway is susceptible to different factors, such as inflammatory mediators and key enzymes, and thus change the relative abundance of metabolic molecules, such as ceramides and S1P, which can alter the follicular cellular environment and affect the growth cycle of the hair and the production of sebum [35, 36]. In addition, the ether lipid metabolic pathway consists of the biosynthesis and catabolism of platelet‐activating factor (PAF) and ether lipids and has a structural function to make membranes less fluid and more rigid, a membrane kinetic function to promote membrane transport and membrane fusion, and a signaling function as a signaling molecule [37, 38, 39], which may affect the stability and signaling of scalp cells and thus scalp health. Notably, PAF in the pathway is a potent pro‐inflammatory mediator that induces platelet aggregation, binds to receptors to further trigger inflammation, and stimulates the production and release of other inflammatory mediators [40, 41]. In hair loss and oily scalp problems, this chronic inflammation can lead to follicle damage and affect normal hair growth.

### 4.2 Differences in Scalp Microbial Communities

The results of alpha diversity analysis showed that the species richness and diversity of the scalp microbial communities in the experimental groups of Hangzhou and Beijing were higher than those in the control groups. Whereas microbiome imbalance has been shown to disturb the skin barrier function and then cause related diseases [42], we speculate that changes in the composition of the scalp microbiome can affect the scalp barrier, leading to hair loss and oily scalp problems.

High‐throughput sequencing technology was used for testing in this study. The following conclusions were drawn by analyzing the differences between Hangzhou and Beijing scalp samples at the phylum and genus levels. Decreases in the abundance of *Actinobacteria*, the major taxonomic unit of bacteria including the common scalp microorganism *Cutibacterium* spp. [21, 43], may have an impact on the scalp microbial environment, which may negatively affect scalp health. *Pseudomonas* is a group of opportunistic pathogens that have previously been found to be strongly associated with scalp psoriasis and positively correlated with psoriasis severity [44, 45], suggesting that an increase in the relative abundance of *Pseudomonas* in the scalp compromises scalp health. *Bacillota* has been implicated in the pathogenesis of atopic dermatitis [46], suggesting that changes in its relative abundance can have an impact on the skin barrier, from which we can hypothesize that it is the abnormal proliferation of *Bacillota* that causes impaired scalp barrier function and affects hair loss. *Basidiomycota* is a major fungal phylum, and changes in its relative abundance in the scalp may lead to changes in the abundance of a variety of fungi, such as *Malassezia*, and impact on scalp health, and it is strongly associated with many scalp disorders such as folliculitis [47].

At the genus level, *Cutibacterium* and *Escherichia* were the dominant bacteria screened in both the experimental and control groups. *Cutibacterium* is a lipophilic opportunistic pathogen closely associated with diseases such as acne and AGA, which regulates sebum synthesis, induces inflammatory responses, and inhibits mutual proliferation with *Staphylococcus surfaces* [48, 49, 50]. A decrease in its relative abundance may affect scalp lipid metabolism and cause scalp inflammation, and may also lead to the excessive proliferation of *surface staphylococcus bacteria*, which may disrupt the micro‐ecological balance of the scalp [22]. *Escherichia* is normally found in the human gut [51]. Gut microorganisms have immunomodulatory effects on the skin, and their abnormalities cause an inflammatory response in the skin [52, 53]. The relative abundance of *Escherichia* in the scalp of the problematic population fluctuated abnormally, and we hypothesized that its dysregulation may have caused scalp inflammation and further affected hair loss. *Malassezia* is a dominant fungus screened in the experimental and control groups. *Malassezia*, a lipophilic yeast closely associated with seborrheic dermatitis and dandruff, hydrolyzes sebaceous triglycerides and releases unsaturated fatty acids that affect the scalp environment, which can cause oxidative damage to the scalp and inflammatory skin reactions, and changes in its relative abundance have been documented to impact scalp health [54, 55, 56]. In addition, we detected *Gilliamella*, a bacterium commonly found in the intestinal tract of crown honey bees, only in the scalp of the problematic population, which is oxidative stress tolerant and plays a regulatory role in inflammatory environments [57, 58]. The relationship between *Gilliamella* and scalp microecology and even hair loss has not been clarified, and we hypothesize that it may affect scalp health by producing specific metabolites or interacting with other scalp microbes.

By LEfSe analysis, we screened for intergroup biomarkers of microorganisms specifically distributed in different groups. The reappearance of *Gilliamella* and *Cutibacterium* coincided with our previous analysis, suggesting that these two groups of microorganisms have an important potential role in hair loss and oily scalp problems. In addition, we screened the problematic population for *Klebsiella* as a scalp species biomarker, an opportunistic pathogen present in the human gut [59]. Based on the existing research on the interplay between intestinal dysbiosis and skin homeostasis [53], we hypothesized that *Klebsiella* may trigger or exacerbate the inflammatory response in the scalp, which in turn causes hair loss. In addition, species biomarkers with high relative abundance and significant differences in Hangzhou could be detected among different groups, while only the control group could detect them in Beijing, which may be related to environmental factors and lifestyle factors. Future research can consider simultaneously incorporating environmental data collection and other means for comprehensive analysis to further understand the influencing mechanism.

### 4.3 Interaction and Synergy Between Scalp Lipids and Scalp Microorganisms

There is a close relationship between scalp microbes and scalp lipids, which interact with each other and in turn synergistically influence the physiological state of the scalp. According to the experimental results, there were some differences between the differential microorganisms and differential lipids screened in Beijing and Hangzhou, but the differential microorganisms and differential lipids in the two regions were highly correlated and intersected. Compared with the control group, the relative abundance of *Actinobacteria* and *Cutibacterium* decreased, and the relative abundance of *Pseudomonas* increased in the scalp of both geographic problem groups. This may be because the growth of *Cutibacterium*, which belongs to the phylum *Actinobacteria*, is inhibited by higher concentrations of sebum, and the lipase activity of *Cutibacterium* is also inhibited by unsaturated fatty acids such as DG (18:1_20:3) and TG (16:0_18:2_18:3) [60]. Sphingolipids could induce *Pseudomonas* to secrete ceramidase to cleave ceramides and obtain fatty acids that could be used as a carbon source for *Pseudomonas*, which was beneficial to the growth of *Pseudomonas* [61, 62].

The different trends in the relative abundance of *Bacillota* and *Escherichia* in the two locations could be attributed to the fact that their growth was affected by the ambient temperature and humidity, which were quite different between Beijing and Hangzhou [63, 64, 65]. As intestinal microorganisms, both microorganisms can play a regulatory role in human lipid metabolism [66, 67, 68]. In addition, the relative abundance of *Malassezia*, which belongs to the phylum *Basidiomycota*, was higher in skin exposed to more polluted air [69], and the higher air pollution in Beijing compared to Hangzhou may be one of the reasons for the different trends in relative abundance of both *Basidiomycota* and *Malassezia* measured in two sites. *Malassezia* uses triglycerides as a carbon source and relies on specific lipids, such as diglycerides and sphingomyelins, to maintain the structure and function of cell membranes, and its colonization of the skin is affected by the decrease in ceramide levels [70, 71]. In addition, by jointly analyzing scalp lipid metabolism and scalp microbiota, we screened the top five pairs of scalp microbes and scalp metabolites with the strongest correlations in different regions, and their relationships and synergistic effects on the scalp are still unclear and deserve to be explored in subsequent studies.

### 4.4 Possible Impact of Geographical Differences on Research Findings

Through the analysis, we found obvious geographical differences between Beijing and Hangzhou in scalp physiological parameters, lipid metabolism results, and scalp microbial community structure. In terms of microbial community, there was a certain trend of separation between the experimental group and the control group in Beijing, while the trend of separation was not obvious in Hangzhou. The reasons for these geographical differences are not yet clear, but we speculate that they can be considered from the following aspects: First, environmental factors, Beijing and Hangzhou have different temperatures, humidity, ultraviolet intensity, light time, and other climatic factors, which may lead to different scalp conditions in the two places; second, living habits, Beijing and Hangzhou residents have very different dietary habits; third, genetic factors, residents of Beijing and Hangzhou may have different genetic backgrounds, which may affect the activity of sebaceous glands and hair loss. Changes in lipids and microorganisms in populations between geographic regions due to these causes may indirectly affect the physiological state of the scalp and ultimately affect hair loss.

### 4.5 Limitations and Directions for Future Research

This study still has some limitations that need to be addressed in future studies. First, the study population was relatively limited to young females in Beijing and Hangzhou, so the findings may not be representative of all young females. Future studies could expand the sample size and conduct multicenter studies of young females from different regions and ethnicities to reduce limitations in number, geography, and ethnicity. Second, the existing literature has rarely investigated the relationship between the scalp lipids, lipid metabolic pathways, and scalp microorganisms that we screened for and the problem of hair loss and oily scalp, and future studies could make more attempts in this direction. Third, the associations found between specific scalp lipids and metabolic pathways as well as abnormal scalp microbial activity and hair loss and oily scalp problems cannot be directly inferred as causal relationships in this cross‐sectional, controlled study and need to be further explored in subsequent studies utilizing longitudinal and intervention studies before definitive conclusions can be drawn. In addition, we used a crude visual assessment of overall hair density by a licensed dermatologist in the experimental design, and future studies could use analytical tools that yield more accurate data, such as trichoscopy. Future research can conduct a comprehensive analysis of scalp microbiota and gut microbiota to draw more comprehensive conclusions.

## 5 Conclusion

This study analyzed the differences in scalp physiological indicators, lipid metabolism, and scalp microbiota to reveal the potential associations between these factors and hair loss and oily scalp problems in young Chinese females. Abnormal activities of specific lipids and metabolic pathways, as well as abnormal activities of specific microorganisms, were found to be strongly associated with hair loss and oily scalp problems. Although the results cannot yet prove a direct causal relationship, this study deepens the understanding of the possible causes of hair loss and oily scalp problems in young Chinese females, especially the imbalance of specific scalp lipid metabolism and the changes occurring in specific scalp microorganisms, and provides some reference value for further research. In addition, our findings also offer new ideas for developing corresponding hair and scalp care products, which can be beneficial for patients to improve their scalp condition.

## Author Contributions

**Siqi Shao:** data collection, statistical analysis, and manuscript drafting. **Benyue Li:** methodology, validation, formal analysis, investigation. **Jie Yang:** methodology, validation. **Fengwei Qi:** methodology, validation. **Qi Liu:** methodology, validation. **Fengnian Zhao:** conceptualization, supervision, writing – review and editing.

## Ethics Statement

This study protocol was reviewed and approved by Shanghai Ethics Committee for Clinical Research, approval number SECCR2023‐161‐01.

## Conflicts of Interest

The authors declare no conflicts of interest.

## Supporting information


**Figure S1:** Steps for dividing 12 partitions: confirm the centers of the two ears, and the line connecting them is called the “peak of the ear line”; the center line of the scalp perpendicular to the peak of the ear line is noted as the “half‐way line”; the total length of the center parting line is from the front hairline to the back hairline position, divide the median line into four equal parts, the area bounded by it being designated as areas “A” to “H,” and the left and right sides where the two ears are located are divided into regions “I” to “L” according to the line of the peaks of the ears.
**Figure S2:** Model validation and scoring plots for OPLS‐DA. (A) OPLS‐DA model validation plots for WT‐BJ (Beijing experimental group) and ZC‐BJ (Beijing control group) groups. (B) OPLS‐DA model validation plots for WT‐HZ (Hangzhou experimental group) and ZC‐HZ (Hangzhou control group) groups. (C) OPLS‐DA score plots for groups WT‐BJ and ZC‐BJ. (D) OPLS‐DA score plots for the WT‐HZ and ZC‐HZ groups.
**Figure S3:** Box plots of the distribution of abundance of Metastats significantly differentiated species between different groups. Figures A–J are, in order, *Actinomycetota*, *Bacteroidota, Campylobacterota*, *Thermotogota*, *Chytridiomycota*, *Zoopagomycota, Hofneiviricota*, *Kitrinoviricota*, *Pisuviricota*, and *Fusobacteriota*. In the figure, the horizontal axis shows the sample groupings, the vertical axis shows the relative abundance of the corresponding species. The horizontal lines represent the two subgroups with significant differences, while the absence of a line indicates that the species does not differ between the two subgroups. “*” indicates a significant difference between the two groups (q value < 0.05), and “**” indicates a highly significant difference between the two groups (q value < 0.01).
**Figure S4:** Heat maps of Pearson correlation hierarchical clustering of differential microorganisms and differential metabolites at the genus level in (A) Beijing and (B) Hangzhou (“*” indicates a significant difference between the two groups (*p* value < 0.05), and “**” indicates a highly significant difference between the two groups (*p* value < 0.01)). The Pearson correlation and chordal diagrams of the different microorganisms and metabolites at the genus level in (C) Beijing and (D) Hangzhou.
**Table S1:** Detailed information on the scalp measurement area.
**Table S2:**. Head hair density assessment scale.

## Data Availability

The data that support the findings of this study are available from the corresponding author upon reasonable request.
