# Supplementary material for: Multidimensional Research on Hair Loss in Young Chinese Females With Oily Scalps
Source: J Cosmet Dermatol. 2025 Sep 7;24(9):e70426. doi: 10.1111/jocd.70426 (PMC12414869; doi:10.1111/jocd.70426)
Supplement: Supplementary file 1 — Figure S1: Steps for dividing 12 partitions: confirm the centers of the two ears, and the line connecting them is called the “peak of the ear line”; the center line of the scalp perpendicular to the peak of the ear line is noted as the “half‐way line”; the total length of the center parting line is from the front hairline to the back hairline position, divide the median line into four equal parts, the area bounded by it being designated as areas “A” to “H,” and the left and right sides where the two ears are located are divided into regions “I” to “L” according to the line of the peaks of the ears. Figure S2: Model validation and scoring plots for OPLS‐DA. (A) OPLS‐DA model validation plots for WT‐BJ (Beijing experimental group) and ZC‐BJ (Beijing control group) groups. (B) OPLS‐DA model validation plots for WT‐HZ (Hangzhou experimental group) and ZC‐HZ (Hangzhou control group) groups. (C) OPLS‐DA score plots for groups WT‐BJ and ZC‐BJ. (D) OPLS‐DA score plots for the WT‐HZ and ZC‐HZ groups. Figure S3: Box plots of the distribution of abundance of Metastats significantly differentiated species between different groups. Figures A–J are, in order, Actinomycetota, Bacteroidota, Campylobacterota, Thermotogota, Chytridiomycota, Zoopagomycota, Hofneiviricota, Kitrinoviricota, Pisuviricota, and Fusobacteriota. In the figure, the horizontal axis shows the sample groupings, the vertical axis shows the relative abundance of the corresponding species. The horizontal lines represent the two subgroups with significant differences, while the absence of a line indicates that the species does not differ between the two subgroups. “*” indicates a significant difference between the two groups (q value < 0.05), and “**” indicates a highly significant difference between the two groups (q value < 0.01). Figure S4: Heat maps of Pearson correlation hierarchical clustering of differential microorganisms and differential metabolites at the genus level in (A) Beijing and (B) Hangzhou (“*” in [file JOCD-24-e70426-s001.docx]

***Supplementary Material***

***Multidimensional Research on Hair Loss in Young Chinese Females with Oily Scalps***

1. **Specific Steps for Instrument Operation:**

*1.1 Hydration Operation Process*

First, place the probe perpendicular to the area of the skin to be measured. Next, control the movement of the probe in the test area within 2 cm x 2 cm. Conduct three parallel data collections in the same test area. After completing data collection, check that the maximum difference between the three parallel data sets does not exceed 30 or the RSD does not exceed 25%. If the criteria are met, then the average of the three data sets can be calculated and recorded.

*1.2 Tewameter Tm Nano Operation Process*

First, gently place the probe on the test area, making sure that the probe touches the skin vertically and that there is no gap between the probe and the test area. Then, control the movement of the probe over the test area to a range of 2 cm x 2 cm and start the measurement. When taking measurements, keep the thumb in position to touch the white button on the probe handle at all times while allowing the internal elements of the probe chamber to touch the skin on the lower side. And use the MPA test software to set the Tewameter to calculate the mean and standard deviation (extended threshold) every five measurements, checking that the standard deviation (SD) value is less than 0.15 g/m²/h and that the measurement time is greater than 20 seconds. If the requirements are met, after completing the measurements, the 3 values from the last set of data are recorded and averaged.

*1.3 Skin-pH-Meter PH 905 Operation Process*

First, make the glass probe at the bottom of the test instrument touch the skin vertically in the area to be tested, and then click the test button to start the measurement. Successive measurements are made until the maximum difference between the three measured values is within 0.1. When the requirements are met, the corresponding data are recorded and averaged.

*1.4 Meibometer MB 560 Operation Process*

First, cut the test tape and fix both ends on the test probe so that the frosted side is up, then stick the device vertically on the test site for 30 seconds. The test strip is then removed and secured to the test bench. Then, push the test bench to the bottom of the tester and draw it outward at a constant speed after 1 second to complete the measurement and record the data.

1. **Detailed Process and Software Parameters for Processing Scalp Microbiology Test Results**

First, fastp software was used for raw data quality control. The default parameters of the software were selected to pre-process the raw data (Raw Data) obtained from the Illumina HiSeq sequencing platform, resulting in the valid data (Clean Data) for subsequent analysis. If there was contamination in the sample, it was compared with the host database to filter out reads that may have originated from the host.

After obtaining the Clean Data, assembly analysis was performed using the MEGAHIT assembly software. The assembly parameters are -k-min 35--k-max 95 --k-step 20 --min contig-len 500. The Bowtie2 software was used to compare the Clean Data after quality control of each sample with the contigs of the assembled samples to obtain the unutilized PE reads. The comparison parameters are -I 200, -X 400. The unutilized reads from each sample were then put together and mixed for assembly with the same parameters as the single-sample assembly.

Next, open reading frame (ORF) prediction was performed using MetaGeneMark with default parameters from each sample and mixed assembled contigs (>=500bp). Based on the prediction results, the predicted genes with lengths less than 100nt were filtered out. The ORF prediction results of each sample and mixed assembly were combined, and the redundancy was removed using CD-HIT software to obtain the initial non-redundant gene catalog (here, non-redundant nucleic acid sequences coding for consecutive genes are referred to as genes for operational purposes). Then, the catalog was clustered by default with 95% identity and 90% coverage, and the longest sequences were selected as representative sequences. The parameters used were -c 0.95, -G 0, -aS 0.9, -g 1, -d 0. Then, the Clean Data of each sample was aligned to the initial gene catalogue using Bowtie2, and the number of reads of the genes aligned to each sample was calculated. The comparison parameters used here are --end-to-end, --sensitive, -I 200, -X 400. Finally, the genes supporting reads <= 2 in each sample were filtered out to obtain the final gene catalogue (Unigenes) for subsequent analysis.

The Unigenes sequences were compared with bacterial, fungal, archaeal and viral sequences extracted from NCBI's NR (version: 2022.05) database using DIAMOND software, and the comparison results with an evalue <= 10 times the minimum evalue were selected, and the final species annotation information of the sequences were finally obtained from them by the LCA algorithm. The LCA annotation results were combined with the gene abundance information to obtain the abundance and number of genes at each taxonomic level, and final statistical analyses were performed based on the species abundance statistics table for each level.

1. **Detailed Procedure for Processing and Analyzing Scalp Lipid Samples**

Lipid samples were first removed from the -80°C refrigerator and thawed on ice. After thawing, 6 mL of extraction solvent (MTBE: MeOH = 3:1, v/v) containing the internal standard mixture was added to the original test tube, which was then shaken at 2500 rpm for 15 minutes. The tape was then removed from the test tube using tweezers. Next, 1200 μL of ultrapure water was added, vortexed for 1 minute, and then allowed to stand for 30 minutes at 4°C. The tape was then removed from the tube with tweezers at 3,000 rpm at the same temperature. Then centrifuge at 3,000 rpm for 10 minutes at the same temperature, and collect 2,500 μL of the upper organic layer into the appropriate numbered centrifuge tubes and lyophilize.

Before LC-MS/MS analysis, 200 μL of lipid complex solution was added to the sample. The mixture is vortexed for 3 minutes and then centrifuged at 12,000 rpm for 3 minutes, and the supernatant is collected as a non-conventional sample for further analysis. Sample data was then collected using ultra-performance liquid chromatography (UPLC) and tandem mass spectrometry (MS/MS). The extracts were then analyzed using an LC-ESI-MS/MS system to produce raw data for subsequent analysis.The specific analytical conditions were as follows:

In the UPLC system, the column was Thermo Accucore™ C30 (2.6 μm, 2.1 mm*100 mm i.d.). The solvent system consisted of phase A (60/40, v/v, 0.1% formic acid, 10 mmol/L ammonium formate), consisting of acetonitrile/water, and phase B (10/90, v/v, 0.1% formic acid, 10 mmol/L ammonium formate), consisting of acetonitrile/isopropanol, respectively. According to the gradient program, the volume ratio of phase A to phase B was 80 to 20 at minute 0, 70 to 30 at minute 2, 40 to 60 at minute 4, 15 to 85 at minute 9, 10 to 90 at minute 14, and 5 to 95 at minute 15. The flow rate of the liquid in the system was 0.35 ml/min. The system temperature was 45 °C. The system injection volume was 2 µl. The effluent from the UPLC system was alternately connected to an ESI-triple quadrupole linear ion trap (QTRAP)-MS.

In the QTRAP® LC-MS/MS system, the effluent is passed through a Triple Quadrupole Linear Ion Trap Mass Spectrometer (QTRAP) to obtain LIT and Triple Quadrupole (QQQ) scans. The mass spectrometer is equipped with an ESI Turbo Ion Spray interface and operates in positive and negative ion modes, controlled by Analyst 1.6.3 software (Sciex). Instrument tuning and mass calibration of the effluent were performed using 10 and 100 μmol/L polypropylene glycol solutions in QQQ and LIT modes, respectively. QQQ scans were performed as MRM experiments with the collision gas (nitrogen) set to 5 psi. DP and CE were performed on individual MRM transitions and further optimized for DP and CE. A specific set of MRM transitions was monitored in each period based on the metabolites eluting during this period, culminating in lipid quantification.

The ESI ion source was operated with the following parameters: the ion source was turbojetted. The temperature of the ion source is 500 °C. The ion spray voltages (IS) are 5500 V (positive ions) and -4500 V (negative ions). The ion source gas 1 (GS1), gas 2 (GS2), and curtain gas (CUR) pressures are 45, 55, and 35 psi, respectively, and the collision gas (CAD) in the ion source is medium pressure.


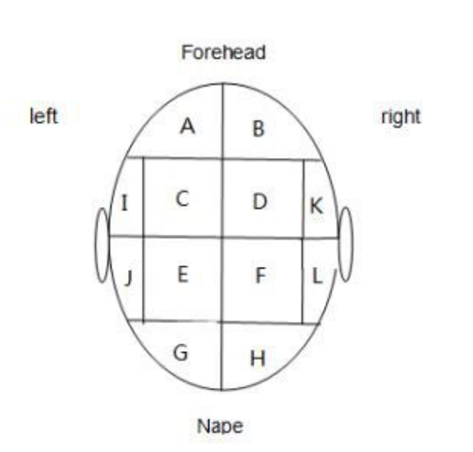


**Fig.S1.** Steps for dividing 12 partitions: confirm the centers of the two ears, and the line connecting them is called the “peak of the ear line”; the center line of the scalp perpendicular to the peak of the ear line is noted as the “half-way line”; the total length of the center parting line is from the front hairline to the back hairline position, divide the median line into four equal parts, the area bounded by it being designated as areas “A” to “H”, and the left and right sides where the two ears are located are divided into regions “I” to “L” according to the line of the peaks of the ears.


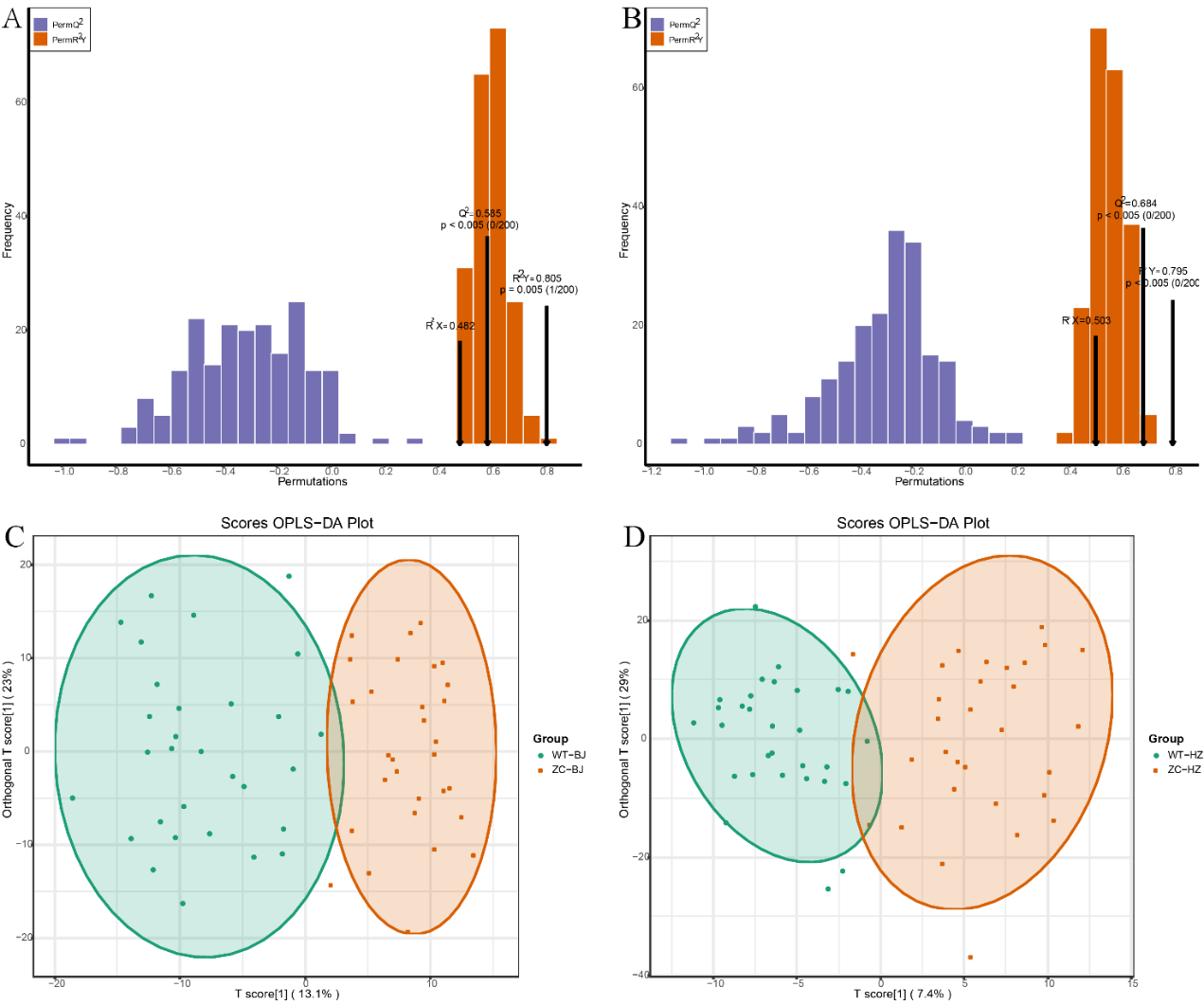


**Fig.S2.** Model validation and scoring plots for OPLS-DA. (A) OPLS-DA model validation plots for WT-BJ (Beijing experimental group) and ZC-BJ (Beijing control group) groups. (B) OPLS-DA model validation plots for WT-HZ (Hangzhou experimental group) and ZC-HZ (Hangzhou control group) groups. (C) OPLS-DA score plots for groups WT-BJ and ZC-BJ. (D) OPLS-DA score plots for the WT-HZ and ZC-HZ groups.


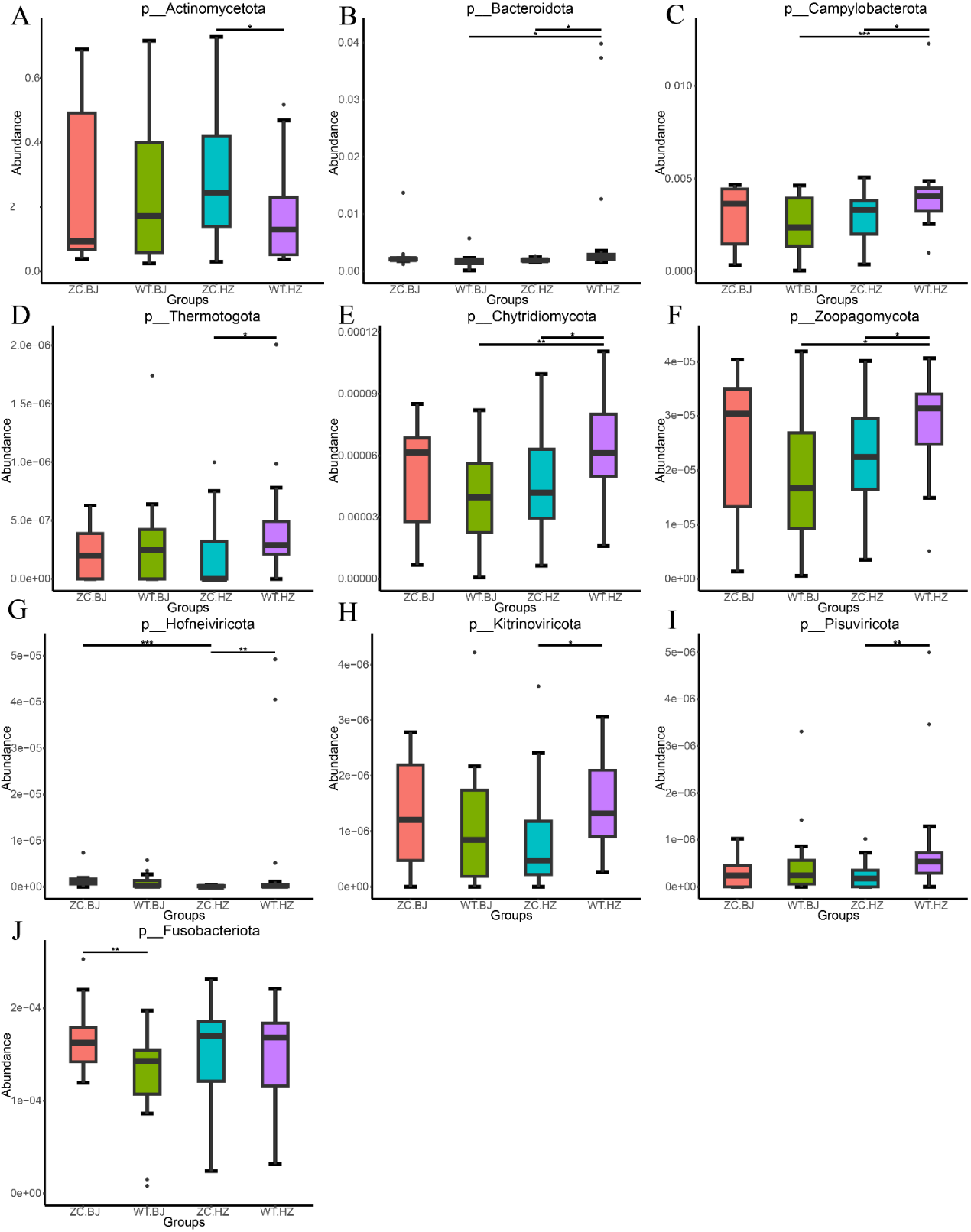


**Fig.S3.** Box plots of the distribution of abundance of Metastats significantly differentiated species between different groups. Figures A-J are, in order, *Actinomycetota*, *Bacteroidota,* *Campylobacterota*, *Thermotogota*, *Chytridiomycota*, *Zoopagomycota,* *Hofneiviricota*, *Kitrinoviricota*, *Pisuviricota*, and *Fusobacteriota.* In the figure, the horizontal axis shows the sample groupings, the vertical axis shows the relative abundance of the corresponding species. The horizontal lines represent the two subgroups with significant differences, while the absence of a line indicates that the species does not differ between the two subgroups. “*” indicates a significant difference between the two groups (q value < 0.05), and ‘**’ indicates a highly significant difference between the two groups (q value < 0.01).


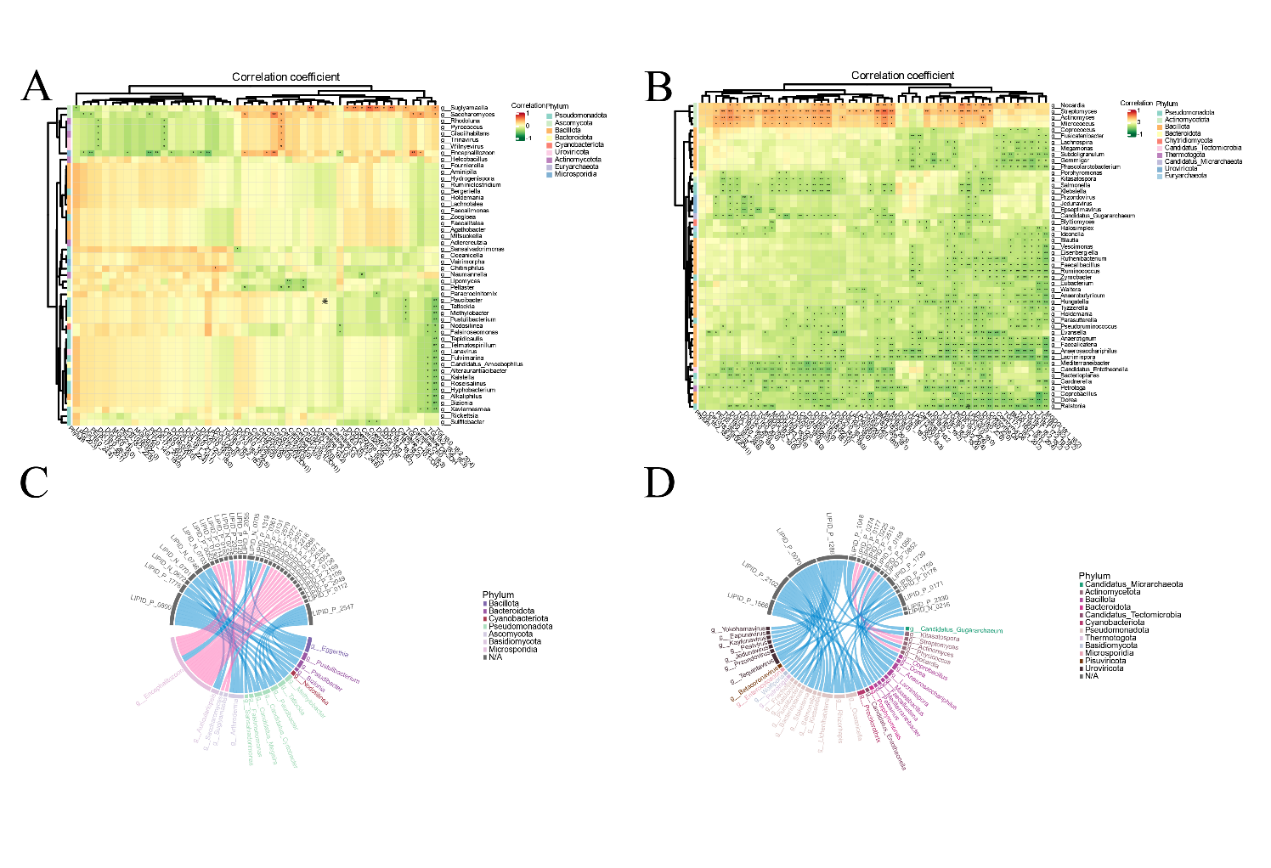


**Fig.S4.** Heat maps of Pearson correlation hierarchical clustering of differential microorganisms and differential metabolites at the genus level in (A) Beijing and (B) Hangzhou (“*” indicates a significant difference between the two groups (p value < 0.05), and ‘**’ indicates a highly significant difference between the two groups (p value < 0.01)). The Pearson correlation and chordal diagrams of the different microorganisms and metabolites at the genus level in (C) Beijing and (D) Hangzhou.

**Table S1.** Detailed information on the scalp measurement area

| Regional name | Specific part |
| --- | --- |
| B | Temple Area |
| C | Side - Head Area |
| D | Above - Ear Area |
| G | Neck - Side Area |
| I | Auricular - Summit Area |
| K | Top - Side Area |

**Table S2.** Head hair density assessment scale

| Grade | Description |
| --- | --- |
| 0 | No hair |
| 1 | Extremely thinning, scalp visible |
| 2 | Thinning, easy to see the scalp |
| 3 | A little sparse, visible scalp |
| 4 | Medium density, small amount of scalp visible |
| 5 | A bit thick, very little scalp visible |
| 6 | Thickness, scalp faintly visible |
| 7 | Very thick, almost invisible on the scalp |
